# Systemic Mastocytosis in a Patient with BCR-ABL1-Positive Chronic Myeloid Leukemia in the Remission Phase

**DOI:** 10.1155/2022/7251658

**Published:** 2022-11-03

**Authors:** Christos Fokoloros, Periklis Foukas, Nikolaos Georgakopoulos, Zoi Tsakiraki, Anthi Bouchla, Vasilliki Pappa, Alexandros Katoulis, Michael Makris, Sotirios Papageorgiou

**Affiliations:** ^1^Allergy Unit “D. Kalogeromitros”, 2nd Department of Dermatology and Venereology, Medical School, National and Kapodistrian University of Athens, Attikon University Hospital, Athens, Greece; ^2^2nd Department of Pathology, Medical School, National and Kapodistrian University of Athens, Attikon University Hospital, Athens, Greece; ^3^Department of Cytogenetics and Molecular Pathology, Locus Medicus S.A., Athens, Greece; ^4^Hematology Unit, 2nd Department of Internal Medicine, Propaedeutic, Medical School, National and Kapodistrian University of Athens, Attikon University Hospital, Athens, Greece

## Abstract

Systemic mastocytosis (SM) comprises a group of rare disorders resulting from tissue infiltration by pathological mast cells. In a percentage ranging from 5 to 40% in various patient series, SM appears to be associated with an accompanying hematologic neoplasm (SM-AHN). The coexistence of SM with chronic myelogenous leukemia (CML) is extremely rare with only 3 cases in the literature. The natural course of CML has changed dramatically over the past 2 decades with the use of tyrosine kinase inhibitors (TKIs). We report a case of diagnosing SM in a patient in complete molecular remission of CML after stopping TKI treatment.

## 1. Introduction

Systemic mastocytosis (SM) is a rare myeloid neoplasm. According to current guidelines [1], SM is classified into subgroups mainly based on clinical and laboratory findings. Among them, SM with an associated hematologic (non-MC) neoplasm (SM-AHN) was the second most common SM subgroup in data of the European Competence Network on Mastocytosis (ECNM) registry [2].

Chronic myeloid leukemia is a pluripotent stem cell disease characterized by the presence of the Philadelphia chromosome that fuses the BCR gene on chromosome 22 with the ABL1 gene on chromosome 9. The treatment of CML has changed dramatically in the last two decades. According to current guidelines, treatment-free remission is achieved when a patient who has discontinued TKI therapy maintains a deep molecular response (DMR) and does not need to restart treatment [3]. We describe for the first time a case of SM in a patient with CML in the TFR phase.

## 2. Case Report

A 45-year-old man with a personal history of three episodes of near-fatal anaphylaxis after Hymenoptera stings during the last decade was referred to mastocytosis outpatient clinic due to elevated serum tryptase (27.5 ng/mL). The allergological workup detected IgE sensitization to multiple species of Hymenoptera venoms; *Apis mellifera*, *Vespa crabro*, and *Polistes dominula*. The bone marrow (BM) biopsy revealed multifocal aggregates of spindled CD117+, CD2+, CD25+, and CD30+ mast cells, with more than 15 cells per aggregate. Additional studies on the BM samples showed a normal karyotype (46, XY), while allele-specific polymerase chain reaction for KITD816V detection was positive. Therefore, the diagnosis of SM was documented, according to the recent WHO criteria [1].

**Figure 1:**
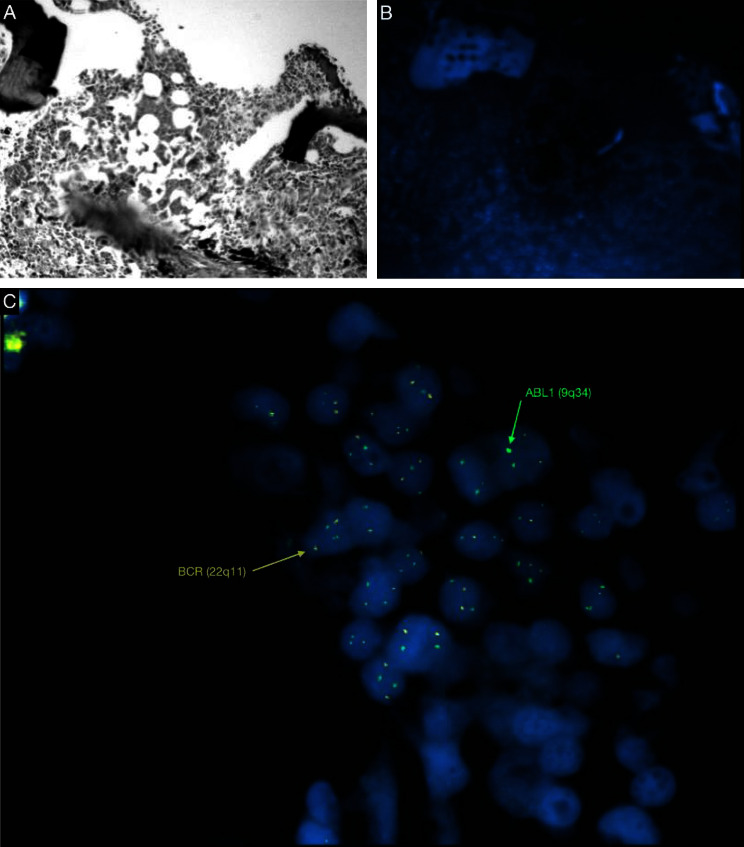
(a): IHC (×10), (b): FISH (×10), and (c): FISH (×100); a fluorescence in situ hybridization (FISH) technique for the detection of the *t* (9; 22) (*q*34; *q*11) reciprocal translocation was performed in samples with mast cell infiltration and was negative.

The patient reported a diagnosis of chronic myeloid leukemia (CML) BCR-ABL-positive (Ph+), 8 years ago. He was treated with dasatinib (100 mg per day), a second-generation TKI, for almost 7 years with full remission (MR 4.5) after 4 to 5 years of therapy and with no complications during treatment. In the last 16 months, the patient has discontinued TKI therapy and maintains a deep molecular response (treatment-free remission/TFR). According to his history, an additional BM study with a fluorescence in situ hybridization (FISH) technique for the detection of the *t* (9; 22) (*q*34; *q*11) reciprocal translocation was performed in samples with mast cell infiltration and was negative (Figure 1). Bone marrow samples from the initial diagnosis of CML were not available to investigate the presence of mast cell infiltration.

The patient's CML remains in a deep molecular response (MR 4.5) and venom immunotherapy has been initiated in a combination of omalizumab (300 mcg per month) due to anaphylactic episodes during immunotherapy. No other cytoreduction therapy for SM was initiated as the patient does not present any c-finding.

## 3. Discussion

Up to now, only three cases of SM with associated Ph+ CML have been reported in the literature [4–6]. In our case, the first episode of anaphylaxis occurred before the diagnosis of CML, so we could assume that SM was preexisting. In the other two reported cases of SM-CML, the concomitant SM was present from the initial time point of CML diagnosis [4, 5].

Hymenoptera venom allergy is a typical IgE-mediated disease, in which clinical manifestations are the result of mast cells' (MCs) degranulation. The prevalence of venom allergy in SM patients is higher than in the general population and venom sting represents the most common trigger of anaphylaxis in adult mastocytosis patients. Also, non-IgE degranulation of MCs may occur in patients with SM [7]. A clinical history of multiple near-fatal anaphylactic episodes is strong evidence for additional investigation, especially in a patient with already known myeloid neoplasm.

In conclusion, in this case, the coexistence of two rare genetic aberrations drives the development of two distinct neoplastic diseases, for which the targeted treatment of CML had no effect on the natural history of the SM.

## Data Availability

The data used to support the findings of this study are included within the article.
